# Supplementary material for: Parenchymal border macrophages regulate tau pathology and tau-mediated neurodegeneration
Source: Life Sci Alliance. 2023 Aug 10;6(11):e202302087. doi: 10.26508/lsa.202302087 (PMC10415611; doi:10.26508/lsa.202302087)
Supplement: Supplementary file 1 [file LSA-2023-02087_TableS1.docx]

Sex effect						
Analysis	Figure	% of total variation	P value	P value summary	Significant?	Comments
% OVA coverage	1.c.	2,554	0,4448	ns	No	
% OVA coverage	1.e.	0,6218	0,61	ns	No	
PBM numbers	1.g.	11,24	0,1685	ns	No	
MHCII+CD38- PBMs		2,425	0,5017	ns	No	
MHCII+CD38+ PBMs		3,964	0,3798	ns	No	
MHCII-CD38+ PBMs		9,997	0,2083	ns	No	
cd206 volume in HC	2.c.	0,1913	0,7556	ns	No	
% AT8 in HC	2.g.	11,56	0,0187	*	Yes	
% AT8 in Pir/Ent CTX	2.h.	2,961	0,2342	ns	No	
% PG5 in HC	2.i.	0,006068	0,9568	ns	No	
% PG5 in Pir/Ent CTX	2.j.	0,2062	0,7512	ns	No	
% MC1 in HC	2;K	0,5694	0,5955	ns	No	
% MC1 in Pir/Ent CTX	2.l.	0,1502	0,791	ns	No	
Insoluble pTau	2.m.	5,49	0,1079	ns	No	
Soluble pTau	2.n.	3,161	0,2241	ns	No	
Insoluble total tau	2.o.	3,348	0,2199	ns	No	
Soluble total tau	2.p.	0,4267	0,6728	ns	No	
Volume HC	2.r.	0,005102	0,9563	ns	No	
Volume Pir/Ent CTX	2.s.	15,09	<0,0001	****	Yes	Interaction p<0.0001
DG layer thickness	2.u.	0,01042	0,9407	ns	No	
Iba 1 volume	3.b.	7,684	0,0583	ns	No	
Clec7a volume	3.c.	1,287	0,4325	ns	No	
P2ry12 volume	3.d.	1,292	0,4153	ns	No	
GFAP volume	3.e.	5,806	0,1024	ns	No	
AQP4	4.b.	5,379	0,1194	ns	No	
Depolarized AQP4	4.c.	3,299	0,2254	ns	No	
tyrobp	3.f.	1,799	0,4181	ns	No	
lyz2		4,367	0,207	ns	No	
b2m		0,7023	0,6235	ns	No	
ctsd		0,7114	0,605	ns	No	
cst7		1,783	0,4257	ns	No	
clec7a		0,01727	0,9386	ns	No	
itgax		2,688	0,3181	ns	No	
CD68		1,294	0,4984	ns	No	
trem2		3,017	0,2967	ns	No	
cd9		1,04	0,5417	ns	No	
hexb		3,447	0,2706	ns	No	
cx3cr1		1,269	0,4925	ns	No	
tmem119		1,026	0,555	ns	No	
axl		3,363	0,2597	ns	No	
apoe		0,06553	0,8733	ns	No	
csf1		2,905	0,301	ns	No	
Lamp1		0,08128	0,8629	ns	No	
p2ry12		0,02714	0,9239	ns	No	
lcn2		0,3726	0,7216	ns	No	
hspb1		1,196	0,5135	ns	No	
cxcl10		0,001127	0,9842	ns	No	
cd44		0,2503	0,7609	ns	No	
vim		0,6362	0,6317	ns	No	
gfap		1,171	0,5085	ns	No	
s100a10		2,088	0,3997	ns	No	
cd109		0,09881	0,8526	ns	No	
cd14		3,018	0,2944	ns	No	
aldh1l1		12,49	0,018	*	Yes	Interaction p=0.0298
Mt1		3,582	0,2534	ns	No	
Mt2		5,336	0,1577	ns	No	
Clu		0,05919	0,8842	ns	No	
Dbi		0,5863	0,6534	ns	No	
Fxyd1		0,0002052	0,9931	ns	No	
Prdx6		1,74	0,4015	ns	No	
S100B		2,898	0,3051	ns	No	
Gpc5		3,235	0,2814	ns	No	
Wdr17		1,522	0,4723	ns	No	
il1b		2,247	0,3814	ns	No	
il6		0,2022	0,7973	ns	No	
tnf		0,005754	0,965	ns	No	
ccl2		4,469	0,2071	ns	No	
tgfb		5,701	0,1453	ns	No	
ccl6		2,485	0,3369	ns	No	
cxcl1		0,2249	0,7847	ns	No	
Irf8		1,662	0,4399	ns	No	
Abca1		0,2868	0,7231	ns	No	
C1qa		3,12	0,2845	ns	No	
itgam		2,587	0,3398	ns	No	
C3		0,6928	0,6122	ns	No	
aqp4		0,3489	0,7247	ns	No	
Bace2		0,06595	0,8785	ns	No	
Mapt		0,794	0,597	ns	No	
syp		0,365	0,7242	ns	No	
TLR4		2,13	0,3897	ns	No	
TLR2		2,401	0,3563	ns	No	
fcgr3 (CD16)		2,697	0,3269	ns	No	
CSF3R (cd114)		3,037	0,2972	ns	No	
